# Case Report: Cariprazine Efficacy in Young Patients Diagnosed With Schizophrenia With Predominantly Negative Symptoms

**DOI:** 10.3389/fpsyt.2021.786171

**Published:** 2021-11-22

**Authors:** Octavian Vasiliu

**Affiliations:** Department of Psychiatry, Dr. Carol Davila University Emergency Central Military Hospital, Bucharest, Romania

**Keywords:** novel atypical antipsychotics, negative symptoms, schizophrenia, cariprazine, tolerability, quality of life, social reintegration

## Abstract

Negative symptoms of schizophrenia are among the most invalidating clinical manifestations of this disorder, and they are correlated with poorer prognosis, lower quality of life, and fewer chances for successful social reintegration and professional rehabilitation. Although atypical antipsychotics have been associated with higher efficacy on negative symptoms than typical agents, not all of them are equally effective. Cariprazine is a new D3 and D2 receptor partial agonist, and its high D3 affinity may be useful for decreasing several adverse events (e.g., extrapyramidal symptoms or hyperprolactinemia), and also for increasing this drug's efficacy over negative symptoms. This case series presents three young adults with predominantly negative symptoms during treatment with an atypical antipsychotic, administered in stable dose within the therapeutic range, and for at least 4 weeks prior to the cariprazine switch. These patients (two male and one female, mean age 35.7 years) were diagnosed with schizophrenia, according to the DSM-5 criteria. They were evaluated using Positive and Negative Syndrome Scale (PANSS), Clinical Global Impression-Severity (CGI-S), and Global Assessment of Functioning (GAF). Their mean initial values were 80.3 on PANSS, 4.3 on CGI-S, and 48 on GAF. All these patients were already on a treatment with stable doses of atypical antipsychotics (olanzapine 10 mg/day, *n* = 1, risperidone 6 mg/day, *n* = 1, and quetiapine 600 mg/day, *n* = 1). Cross-titration to cariprazine was initiated, from 1.5 mg qd up to 6 mg qd, during a mean period of 2.7 weeks. After 12 weeks of cariprazine 6 mg/day, the positive scale of PANSS was relatively stable compared to baseline, while the negative mean score decreased by 22%. Also, the mean CGI-S improvement was 15.4% and the GAF mean score increased by 17%. The overall tolerability was good, without severe adverse events being reported. Conclusions: Cariprazine is well tolerated and efficient for patients diagnosed with schizophrenia who have significant negative symptoms that impair daily functioning. After 12 weeks cariprazine succeeded in improving negative symptoms, global functioning, and clinical global impression.

## Introduction

Negative symptoms of schizophrenia are among the most invalidating clinical manifestations of this disorder, and they are correlated with poorer prognosis, lower quality of life, and fewer chances for successful social reintegration and professional rehabilitation (1–3). Targeting negative symptoms (e.g., apathy, alogia, flat affect) can lead to significant improvements of daily functional and quality of life (4). Although atypical antipsychotics have been associated with higher efficacy over the negative symptoms than typical agents, not all the atypicals are equally effective. According to a meta-analysis of placebo-controlled and head-to-head randomized controlled trials (*n* = 402 studies, *N* = 53,463 participants) that compared 32 antipsychotics, only clozapine, amisulpride, olanzapine, and, to a lesser degree, zotepine and risperidone decreased negative symptoms severity more than other agents, while the differences between the remaining drugs were less supported by evidence (5). An important problem that may lead to uncertainty in the interpretation of negative symptoms improvement in clinical trials is represented by lack of discrimination using standard measurements between primary and secondary negative symptoms (6). Therefore, the clinician should address this problem during the psychiatric interview, and to take into account any other sources of information available (medical personnel, family members or other caregivers), in order to differentiate between primary and secondary negative symptoms. This is not of scholastic importance, but it has practical utility, due to the different treatment approaches for the two groups of symptoms.

Cariprazine is a new D3 and D2 receptor partial agonist, and its high D3 affinity may be useful for decreasing several dopamine-related adverse events, and, in the same time, for increasing this drug's efficacy over negative symptoms (7). The efficacy and safety of cariprazine have been demonstrated in adults with schizophrenia during four short-term randomized, double-blind, placebo-controlled trials, two long-term open-label studies, one relapse prevention study, and one prospective negative symptom study vs. the active comparator risperidone (8). *Post-hoc* analyses supported efficacy of cariprazine across individual symptoms and domains of schizophrenia, and in areas like cognition, functioning, negative symptoms, hostility, and global well-being (8).

Cariprazine was generally well tolerated in clinical trials in patients with schizophrenia, and the most frequently reported adverse events were of mild to moderate severity (7). Cariprazine may reduce side effects when switching a patient from other antipsychotic because of its lower anticholinergic, anti-adrenergic, antihistaminergic, and metabolic effects, with a better cardiovascular safety profile (9, 10).

In a multicentric, randomized, double-blind, phase 3b trial (*N* = 533 patients with predominant negative symptoms), cariprazine (3–6 mg/day) was superior to risperidone (3–6 mg/day) in leading to significant greater least squares mean change in Positive and Negative Syndrome Scale- factor score for negative symptoms (PANSS-FSNS) after 26 weeks of treatment (11). This trial was well-controlled for secondary negative symptoms, but it was sponsored by the manufacturer of cariprazine (6, 11).

According to the recommendations from an International Panel for the management of schizophrenia, cariprazine is useful in patients with first episode of psychosis, predominant negative symptoms (maintenance/acute phase) and significant side effects (e.g., metabolic syndrome, sedation, hyperprolactinemia) with onset during the administration of other antipsychotics (9). If the weight is placed on the long-term efficacy and tolerability, cariprazine may become one of the first-line medications in schizophrenia, not only for prominent negative symptoms, but also for relatively severe positive symptoms (9). An overlap of at least 2–3 weeks is usually recommended in clinical practice when switching from other antipsychotics to cariprazine, in order to avoid a dopaminergic, antihistaminergic and/or muscarinic rebound (9).

This case series presents three young adults with persistent negative symptoms during treatment with an atypical antipsychotic, administered in stable doses within the therapeutic range and for at least 4 weeks, prior to the cariprazine switch.

## Case Presentation

The first patient was a male, diagnosed with schizophrenia according to the DSM-5 criteria, age 37.5, who received treatment for the last 6 weeks prior to baseline with risperidone 6 mg daily. He was evaluated because of persistent negative symptoms, consisting mainly of anhedonia, alogia and avolition. This patient had a history of schizophrenia of more than 5 years, and received in the past olanzapine (10 mg qd, for almost 2 years) and amisulpride (800 mg daily, for 2 years), to which he responded partially, because several negative symptoms were still present. The patient accused tolerability issues, namely sedation to olanzapine, and extrapyramidal symptoms to amisulpride. The initial psychiatric examination detected residual positive symptoms- ideas of reference, mild suspiciousness and conceptual disorganization, as well as general symptoms- anxiety, insomnia, social withdrawal, poor attention and low memory performances. This patient had no family history of psychiatric disorder and no somatic comorbidity could be identified during the initial visit.

The first evaluation detected a total PANSS score of 80, with a negative scale score of 32, a CGI-S (Clinical Global Impression- Severity) value of 4 and a GAF (Global Assessment of Functioning) score of 52. Cariprazine was initiated based on this antipsychotic pharmacodynamics profile and its presumed efficacy over the negative symptoms. Risperidone was gradually tapered off, while cariprazine was initiated with 1.5 mg and titrated up to 6 mg qd, during a period of 15 days. No incident was reported during the cross-over period.

After 12 weeks of stable dose, the PANSS total score decreased to 66, with the negative scale showing a value of 26, the CGI-S score remained stable, and the GAF score increased to 60. The positive PANSS score decreased minimally, from 21 to 19. This patient reported no adverse events during the 12 weeks of the 6 mg qd cariprazine regimen.

The second patient was a male, age 33.5, diagnosed with schizophrenia for 11 years, who received treatment with olanzapine 10 mg qd for the last 8 weeks. He was previously on treatment with risperidone 8 mg/day for multiple periods of 6–12 months, interrupted by lack of adherence. Also, the patient received treatment with risperidone microspheres, up to 50 mg every 2 weeks, but after more than 1 year he declined the need for any injectable treatment and was switched back on oral medication. This patient had a family history of psychiatric disorder, as his father also had schizophrenia. No somatic comorbidity could be identified during the initial visit.

During the initial psychiatric evaluation this patient presented with fragmentary persecutory delusions without significant behavioral impact and prominent negative symptoms, especially flat affect, avolition, and anhedonia. His baseline PANSS total score was 78, with negative subscale score of 29, positive subscale score of 18, CGI-S score of 4, and GAF score=44. Olanzapine was gradually tapered off, while cariprazine was slowly titrated up to 6 mg qd, during 20 days. No clinical signs of positive or negative symptoms worsening was reported during the titration period.

After 12 weeks of cariprazine administered 6 mg qd, the PANSS total score decreased to 62, with the negative scale showing a value of 22, and the positive scale a value of 16. The CGI-S score decreased to three, while the GAF score improved by eight points, reaching a value of 52.

The third patient was a female, age 36, diagnosed with schizophrenia for 6 years, and she received treatment with quetiapine 600 mg qd for the last month. This patient had no family history of psychiatric disorder and no somatic disease could be identified during the initial visit. She had a personal history of multiple antipsychotics prior to the baseline treatment, including typical (haloperidol, zuclopentixol) and atypical (olanzapine, ziprasidone) agents. Her response to quetiapine was initially good, because it alleviated insomnia and anxiety, but the impact over the negative symptoms was less significant. Therefore, she was switched on cariprazine, starting from 1.5 mg, up to 6 mg qd, during a period of 22 days.

The initial psychiatric evaluation detected mainly negative symptoms, consisting of anhedonia, flat affect, avolition, low attention and memory performances. Her baseline PANSS total score was 83, with negative subscale score of 35 and positive subscale score of 23, CGI-S=5, and GAF=57. After 12 weeks of cariprazine stable dose treatment, PANSS total score decreased to 65, with negative score reaching a value of 27, while the positive score was relatively stable (22, final visit score). The CGI-S score at endpoint was 4, and the GAF improved to 60.

**Figure 1:**
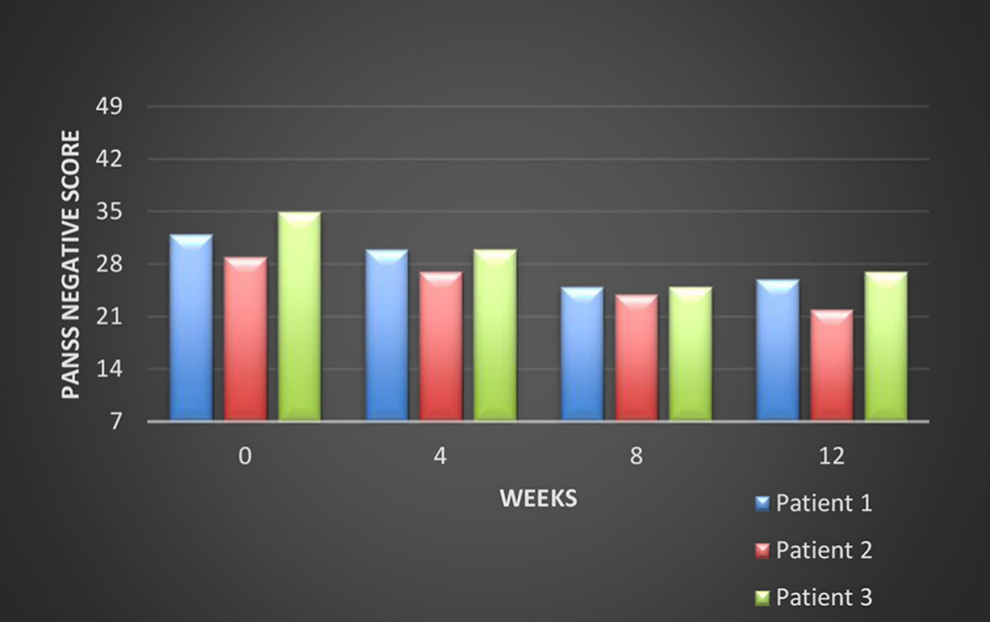
Evolution of the PANSS negative subscale scores during cariprazine treatment.

**Figure 2:**
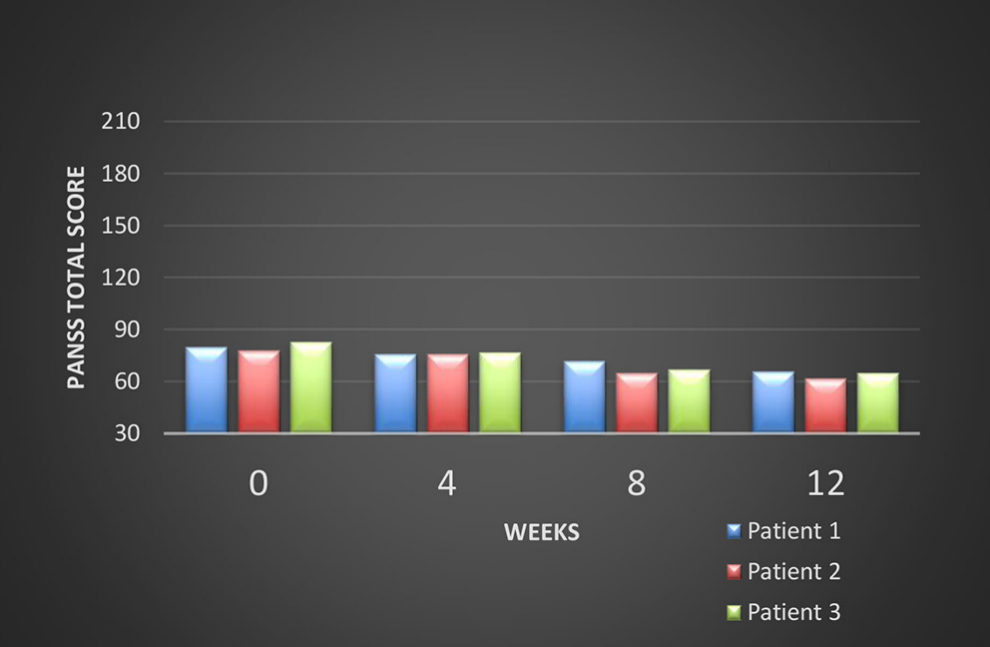
Evolution of the PANSS total scores during cariprazine treatment.

**Figure 3:**
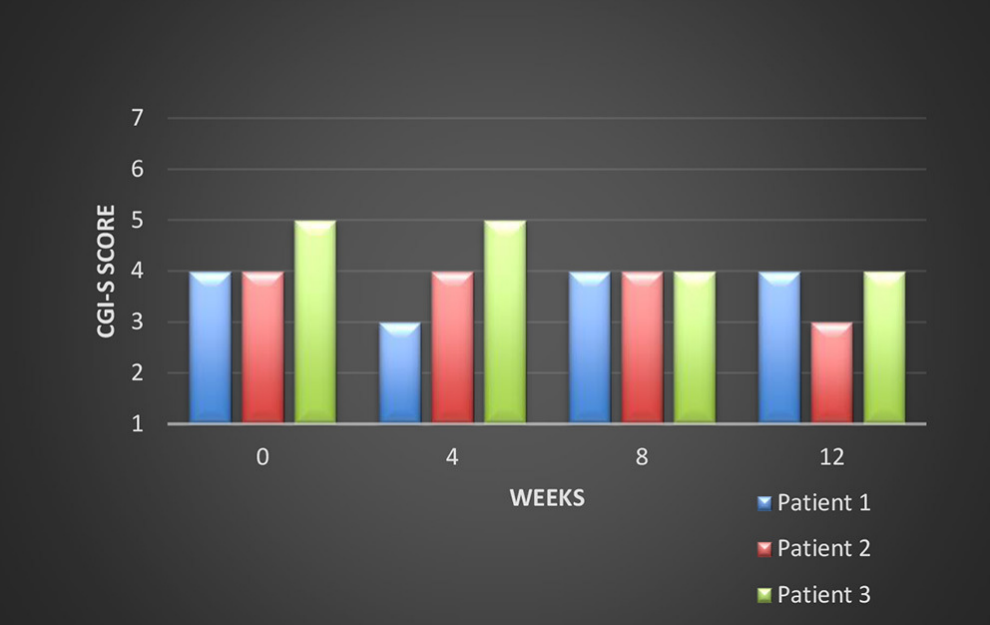
Evolution of the CGI-S scores during cariprazine treatment.

All these patients were screened for psychiatric comorbidities at baseline using Mini-International Neuropsychiatric Interview (MINI), but no specific diagnoses were detected except for schizophrenia. None of them required hospitalization during their switch and up to the final visit. Cross-titration to cariprazine was well tolerated in all cases, and all the other antipsychotics were tapered slowly in order to avoid antihistaminergic/antidopaminergic rebound. After 12 weeks of cariprazine 6 mg/day, the positive subscale of PANSS showed a relatively stable level, but the negative subscale mean score decreased with 22% (Figure 1). The overall PANSS mean score decreased by 19.5% (Figure 2), the CGI-S mean scores improved by 15.4% (Figure 3), while the mean GAF scores increased by 17%. The overall results are presented in Table 1. No severe adverse events was reported throughout the monitoring period.

**Table 1:** The results of the cariprazine switch during 12 weeks of monitoring.

	**Patient 1**	**Patient 2**	**Patient 3**
Previous medication, dosage, and duration of its administration	Risperidone, 6 mg/day, 6 weeks	Olanzapine, 10 mg/day, 8 weeks	Quetiapine 600 mg/day, 4 weeks
Switch to cariprazine duration	15 days	20 days	22 days
PANSS total score- initial visit	80	78	83
PANSS total score- final visit	66	62	65
PANSS Negative scale score- initial visit	32	29	35
PANSS Negative scale score- final visit	26	22	27
PANSS Positive scale score- initial visit	21	18	23
PANSS positive scale score- final visit	19	16	22
CGI-S initial score	4	4	5
CGI-S final score	4	3	4
GAF initial score	52	44	57
GAF final score	60	52	60
Self-reported/clinician-detected severe adverse events throughout the monitoring period	None	None	None

## Discussion

These patients presented a relatively long history of schizophrenia, between 2 and 11 years (mean value 6.3 years), although their mean age was 35.7 years. They all received multiple treatments before the initiation of cariprazine and presented negative symptoms under their current antipsychotic (olanzapine, quetiapine, or risperidone). Cariprazine is a distinctive antipsychotic agent due to its D3-preferential dopamine partial agonism, which make it preferable for patients with prominent negative symptoms. Patients tolerated well the antipsychotic switch from various antipsychotics to cariprazine. In this case series, after 12 weeks cariprazine succeeded in improving negative symptoms, global functioning, and clinical global impression. The positive symptoms were quite stable, but their low level of severity at baseline may have precluded the observation of a therapeutic effect.

Regarding the limitations of this case series, it must be taken into account the short period of monitoring, which may have prevented the observation of other, long-term, treament effects. Also, variables related to the antipsychotic's adverse events were not monitored in a structured manner, as we only collected patients' reports about tolerability and data from clinical exams during each visit. It is also important to mention that patients included in this case series were relatively stable, based on their initial PANSS, GAF, and CGI-S scores, without severe positive or behavioral symptoms and they did not require hospitalization.

## Patient Perspective

“I was unable to take care of myself because I had no energy. No interest, either. And I was feeling scared or even frightened. I feel now I can go outside if I have to do something. I feel less blocked from within” (Patient number 1).

“I feel less tension inside me now than before. My thoughts are more synchronized with what I do… I can watch a TV movie, which I couldn't do before because I was sort of numb” (Patient number 2).

## Data Availability Statement

The original contributions presented in the study are included in the article/supplementary material, further inquiries can be directed to the corresponding author.

## Ethics Statement

Ethical review and approval was not required for the study on human participants in accordance with the local legislation and institutional requirements. The patients/participants provided their written informed consent to participate in this study. Written informed consent was obtained from the individual(s) for the publication of any potentially identifiable images or data included in this article.

## Author Contributions

The author confirms being the sole contributor of this work and has approved it for publication.

## Conflict of Interest

The author declares that the research was conducted in the absence of any commercial or financial relationships that could be construed as a potential conflict of interest.

## Publisher's Note

All claims expressed in this article are solely those of the authors and do not necessarily represent those of their affiliated organizations, or those of the publisher, the editors and the reviewers. Any product that may be evaluated in this article, or claim that may be made by its manufacturer, is not guaranteed or endorsed by the publisher.
